# Dual pathways of generative AI use: role ambiguity and self-efficacy in employee-AI collaboration

**DOI:** 10.3389/fpsyg.2026.1793095

**Published:** 2026-08-14

**Authors:** Qiannan Zhang, Jingyi Zhang, Shan Dong

**Affiliations:** College of Management, Tianjin University of Finance and Economics Pearl River College, Tianjin, China

**Keywords:** employee-AI collaboration, generative AI, role ambiguity, role theory, self-efficacy

## Abstract

**Introduction:**

The growing use of generative AI tools in everyday work improves efficiency but also creates uncertainty about how employees interpret their roles and whether they are willing to collaborate with AI. Drawing on role theory, this study aims to investigate how generative AI (GenAI) use relates to employee-AI collaboration via two distinct pathways: role ambiguity as a hindrance mechanism and role breadth self-efficacy as an enabling mechanism. This study further examines the moderating role of AI literacy in the above relationships.

**Methods:**

Survey data were collected from 541 employees working in Chinese high-technology firms, and structural equation modeling was adopted to test the proposed research model.

**Results:**

The empirical results reveal that GenAI use simultaneously increases role ambiguity and role breadth self-efficacy. Role ambiguity negatively predicts employee-AI collaboration, whereas role breadth self-efficacy exerts a positive effect. AI literacy significantly moderates both pathways: it weakens the positive association between GenAI use and role ambiguity, and strengthens the positive link between GenAI use and role breadth self-efficacy. Furthermore, employee-AI collaboration is positively associated with job performance.

**Discussion:**

These findings extend role theory by verifying that GenAI functions as both a disruptor and an enabler of employees’ role perceptions, and AI literacy serves as a vital boundary condition shaping how employees construct and understand their work roles when adopting GenAI. Practically, organizations are suggested to clarify work role boundaries, foster employees’ role breadth self-efficacy, and deliver systematic AI literacy training to facilitate high-quality human-AI collaboration.

## 1 Introduction

Generative artificial intelligence (GenAI) is reshaping how work is performed across a wide range of industries, driven by core technological advances such as large language models, transformer architectures, and the capacity to generate human-like text, code, and creative content (Brynjolfsson and Mitchell, 2017). These capabilities enable GenAI tools to provide suggestive guidance, generate drafts, and produce outputs that would otherwise require substantial human effort (Glikson and Woolley, 2020).

Recent academic research has increasingly explored GenAI’s potential to enhance workplace productivity and to foster employee engagement, satisfaction, enthusiasm, loyalty, and innovation capabilities (Lin et al., 2025; Prema and Ragini, 2025). Although GenAI has demonstrated remarkable capabilities, often surpassing humans in speed and content generation, its rapid adoption has sparked intense debate about the future of work (Hermann et al., 2025). Critics have highlighted pressing concerns such as privacy vulnerabilities, diminished emphasis on human judgment, inherent bias in automated systems, mistrust of algorithmic decision-making, and the shift from human-to-human collaboration to hybrid human-AI workflows (Zhang W. et al., 2025). Beneath these concerns lies a more fundamental issue: as GenAI takes over tasks that were previously central to employees’ professional identity, employees may become uncertain about the boundaries of their own role and about what is expected of them in an AI-augmented workplace. Empirical evidence indicates that these role-related uncertainties are exacerbating anxiety among workers, fueling digital fatigue and triggering concerns about career relevance in the era of generative AI (Koo et al., 2021). Whether and how employees are willing to collaborate with GenAI at work has therefore become a pressing question. However, the psychological mechanisms underlying employees’ collaboration intentions with GenAI remain insufficiently understood.

The introduction of GenAI necessitates a fundamental shift in how employees perceive their work roles. Research has shown that the balance between human agency and GenAI assistance influences employee well-being, motivation, and performance. A lack of alignment may undermine their effectiveness in fulfilling their duties (Chuang et al., 2025). The advent of GenAI has profoundly disrupted conventional work paradigms, precipitating transformative shifts in how employees approach their tasks (Bag et al., 2021). Individuals may experience a sense of helplessness in maintaining this equilibrium as they become aware of GenAI’s disruptive potential (Premnath and Lohani, 2024). Specifically, GenAI use may trigger two opposing cognitive responses: on one hand, it may blur the boundaries of job responsibilities and create uncertainty about role expectations, leading to role ambiguity; on the other hand, it may expand employees’ perceived capacity to take on broader responsibilities, enhancing role breadth self-efficacy (Delpechitre et al., 2019; Mahlasela and Chinyamurindi, 2020; Suardi and Furinto, 2023). Not all employees, however, respond to GenAI use in the same way. Some employees interpret GenAI as a threat that undermines their role clarity, while others view it as an opportunity to expand their role capabilities. This suggests that individual differences may determine whether employees respond to GenAI with role ambiguity or role breadth self-efficacy. Nevertheless, the mechanisms through which GenAI-induced role perceptions shape employees’ intentions to collaborate with GenAI are not yet fully understood.

Specifically, this study addresses two questions. First, how does GenAI use relate to employee-AI collaboration through role-based cognitive mechanisms? We propose that GenAI use is related to collaboration intentions through two parallel pathways: role ambiguity, which acts as a hindrance mechanism, and role breadth self-efficacy, which acts as an enabling mechanism. Second, building on the observation that employees differ in how they respond to GenAI, what individual difference explains why some employees experience role ambiguity while others develop role breadth self-efficacy? We propose that AI literacy, defined as individuals’ ability to understand, interact with, and evaluate AI systems (Ng et al., 2021), serves as a critical boundary condition that weakens the hindrance pathway and strengthens the enabling pathway.

Although several psychological mechanisms may explain how GenAI use relates to employee-AI collaboration, we focus on role ambiguity and role breadth self-efficacy for three reasons. First, GenAI does not merely assist with task execution; it disrupts the very definition of employees’ professional roles by generating content that was previously central to their identity. This role-based disruption is precisely what role theory is designed to explain. Role theory posits that new technologies primarily influence employees through changes in role perceptions. Unlike trust in AI or AI anxiety, which are general attitudes toward technology, role ambiguity and role breadth self-efficacy directly capture how employees reinterpret their professional identity when GenAI enters the workplace. This role-based reinterpretation is the core theoretical mechanism that distinguishes GenAI from traditional workplace technologies. Second, prior research has identified role ambiguity as a key stressor in technology adoption contexts (van Slooten et al., 2024; Mañas et al., 2018), and role breadth self-efficacy as a critical driver of proactive behaviors (Parker et al., 2006; Syamsudin et al., 2022). However, no study has examined whether GenAI simultaneously triggers both mechanisms. Third, GenAI is unique because it simultaneously disrupts role clarity and enhances role capability. This paradoxical effect is best captured by the dual pathways of role ambiguity and role breadth self-efficacy. This study answers the call for a more nuanced understanding of how AI shapes employees’ psychological states and behavioral intentions. Other mechanisms, such as trust or anxiety, tend to operate in a single direction and cannot capture this duality.

To answer these questions, we draw on role theory (Katz and Kahn, 1978), which posits that new technologies can significantly reshape employees’ role perceptions and subsequent behaviors. We collected survey data from 541 employees at Chinese high-technology firms and tested our conceptual model using structural equation modeling. The remainder of this paper is organized as follows. Section “2 Literature foundation and hypotheses” reviews the theoretical background and develops the hypotheses. Section “3 Materials and methods” describes the methodology. Section “4 Results” presents the results. Section “5 Discussion” discusses the findings, theoretical contributions, and practical implications. Section “6 Limitations and future research” addresses limitations and directions for future research.

## 2 Literature foundation and hypotheses

### 2.1 Generative artificial intelligence (GenAI)

Generative Artificial Intelligence (GenAI) refers to advanced AI systems capable of generating new content, including text, images, code, audio, and other media formats, based on patterns learned from extensive training data (Bankins et al., 2024; Liu et al., 2025). The focal point of this technological transformation is large language models (LLMs), sophisticated neural network architectures that can generate coherent, contextually relevant, and human-like responses to user prompts (Brynjolfsson and Mitchell, 2017). GenAI has demonstrated unprecedented capabilities in understanding context, generating creative content, answering complex questions, and even exhibiting incipient reasoning abilities that were previously considered exclusive to human intelligence (Sun L. et al., 2025). In this study, GenAI is defined as artificial intelligence systems that generate novel ideas, solutions, and knowledge in response to employees’ inputs.

GenAI has been shown to offer employees significant advantages, including enhanced operational efficiency, improved decision-making accuracy, and the ability to generate valuable insights from complex data (Schiavo et al., 2024). For example, GenAI systems can automate routine cognitive tasks such as email drafting and report summarization, while simultaneously augmenting human capabilities by providing creative suggestions, data-driven recommendations, and analytical support (Bewersdorff et al., 2025). Moreover, GenAI can generate creative content for various professional functions, overcoming limitations in time, expertise, or cognitive bandwidth (Wu et al., 2025). Consequently, GenAI has emerged as a strategic priority for organizations seeking to drive business value, enhance innovation, and maintain competitive advantage in increasingly data-driven markets (Li et al., 2026).

### 2.2 Employee-AI collaboration

Employee-AI collaboration refers to employees’ perceived engagement in collaborative behaviors with AI systems, including AI’s participation in decision-making, forecasting, problem-solving, and information evaluation (Anthony et al., 2023). Unlike traditional automation where AI replaces human labor, GenAI collaboration requires employees to remain actively engaged throughout the process. The AI produces drafts, suggestions, or analyses, while the employee retains control over final outputs, evaluation, and refinement (Wu et al., 2025). This collaborative model features iterative interaction, where employees prompt, query, and critique the AI’s outputs, thereby co-creating solutions that neither could achieve alone.

For employees, human-AI collaboration offers tangible benefits that directly contribute to their work performance. When GenAI systems serve as cognitive partners and analytical assistants, they can quickly generate drafts, explore multiple solutions, and refine outputs through iterative dialogue, thereby reducing the cognitive burden of starting from scratch while maintaining control over final results. Empirical evidence indicates that GenAI assistance significantly improves employee task performance by fostering innovative work behavior, particularly under conditions of task complexity (Zhang W. et al., 2025). Moreover, employee-AI collaboration has been found to reduce workload pressures, which in turn enables employees to take on additional responsibilities and engage in more proactive work behaviors (Sun C. et al., 2025). By freeing employees from repetitive or cognitively draining tasks, GenAI collaboration allows individuals to focus on higher-order activities that require human judgment, creativity, and emotional intelligence, thereby enhancing overall job performance. Accordingly, we propose:

H1: Employee-AI collaboration is positively correlated with their job performance.

### 2.3 Dual pathways from role theory: role ambiguity and role breadth self-efficacy

Role theory was initially introduced as a theoretical framework to elucidate the mechanisms through which employees’ perceptions of their roles influence organizational outcomes. Theorists have argued that employees are constantly subjected to an array of expectations from their work environment, which define their perceptions of their organizational roles. Role theory provides a foundational lens for understanding how environmental changes, such as the use of GenAI, reshape employees’ perceptions of their work roles and subsequently influence their behaviors (Katz and Kahn, 1978; Barley, 1990). According to the theory, employees hold multiple expectations about their professional responsibilities. When these expectations become unclear, inconsistent, or inadequately defined, role ambiguity arises. Conversely, when employees feel confident in executing broader, more proactive tasks beyond their formal job descriptions, they exhibit high role breadth self-efficacy. In the context of GenAI use, both phenomena can be triggered simultaneously, creating two parallel pathways that affect employees’ willingness to collaborate with AI systems.

*Role ambiguity as a hindrance pathway*. Unlike traditional workplace technologies, which have clearly defined functional boundaries (e.g., spreadsheet software performs calculations), GenAI produces content that overlaps with employees’ core cognitive and creative tasks. This overlap blurs the distinction between tasks that belong to the employee and tasks that can be delegated to AI, creating a unique condition for role ambiguity to emerge. Based on role theory, role ambiguity occurs when goals and procedures are unclear or ill-defined, preventing employees from fully comprehending the expectations associated with their professional responsibilities (Katz and Kahn, 1978). For some employees, the introduction of GenAI disrupts this clarity, leading to ambiguity in role enactment.

Moreover, when GenAI outputs conflict with employees’ own professional judgment, expectations, or work standards, role ambiguity may also emerge. Zhou et al. (2025) argued that GenAI’s ability to mimic cognitive, creative, and interpersonal capabilities challenges traditional human-machine boundaries and psychologically threatens workers’ needs for competence, autonomy, and relatedness, directly inducing role ambiguity. Zhao et al. (2025) further showed that the introduction of AI into the workplace triggers jurisdictional conflicts, forcing professionals to engage in boundary struggling and boundary retreating, as they struggle to define whose judgment should prevail when AI output diverges from human expertise. Jolly et al. (2026) also demonstrated that working with AI leads individuals to displace responsibility onto the technology, eroding their sense of personal accountability and blurring decision authority. Based on these findings, we propose the following hypotheses:

H2: GenAI is positively correlated with employees’ role ambiguity.

Research consistently shows that ambiguous role expectations adversely affect employees’ work outcomes, including lower job satisfaction, reduced organizational commitment, and decreased proactive behavior (Eatough et al., 2011; Mañas et al., 2018). Role theory posits that role ambiguity deprives employees of clear behavioral guidance, which reduces their confidence in engaging in role-extending behaviors. Employee-AI collaboration requires employees to proactively integrate AI into their work processes, a behavior that demands clear role expectations. When employees are uncertain about their role boundaries, they are likely to withdraw from such proactive engagement to avoid role conflict or performance evaluation risks (Schiavo et al., 2024).

Complementing this, Bankins et al. (2024), in their comprehensive multilevel review of AI in organizations, identified role ambiguity as a key mechanism through which GenAI use triggers negative employee responses; employees who are uncertain about their evolving role responsibilities tend to withdraw from proactive engagement with AI, viewing it as a threat to their professional identity rather than a collaborative tool. Similarly, Wu et al. (2025) reported that unclear role expectations in AI-augmented work environments lead employees to perceive collaboration with AI as cognitively taxing and psychologically risky, reducing their willingness to engage in joint problem-solving with intelligent systems. Collectively, these findings suggest that role ambiguity undermines employee-AI collaboration by draining psychological resources, heightening risk perceptions, and fostering withdrawal tendencies. Therefore, we propose the following hypothesis:

H3: Role ambiguity is negatively correlated with employee-AI collaboration.

*Role breadth self-efficacy as an enabling pathway.* According to role theory, role breadth self-efficacy reflects employees’ confidence in taking on broader, more proactive tasks beyond their formal job descriptions (Parker, 1998). While role theory identifies this construct, social cognitive theory explains how it develops: self-efficacy is shaped by four sources: mastery experiences, vicarious learning, verbal persuasion, and physiological states. In the context of GenAI use, each of these four sources can be activated. Specifically, GenAI provides mastery experiences when employees successfully use it to complete tasks that were previously beyond their skill set; it offers vicarious learning opportunities as employees observe and internalize novel problem-solving strategies from AI-generated outputs; it delivers verbal persuasion through immediate, actionable suggestions and guidance; and it fosters positive physiological states by reducing cognitive load and alleviating anxiety associated with unfamiliar tasks.

When employees successfully use GenAI to complete tasks that were previously beyond their immediate skill set, they acquire direct evidence of their expanded capability. This positive mastery experience, in turn, strengthens their confidence in taking on even broader responsibilities (Mengmeng, 2024). Moreover, by automating routine cognitive tasks such as drafting, summarizing, or code generation, GenAI alleviates the burden of low-level work, allowing employees to gain more cognitive bandwidth for strategic thinking, creative problem-solving, and role expansion (Sun C. et al., 2025). In addition, employees can internalize new problem-solving strategies and creative approaches by observing how GenAI generates novel solutions. According to Ding et al. (2026), this observational learning provides vicarious experiences that enhance employees’ belief in their own ability to perform similar tasks independently. Finally, when facing unfamiliar or complex tasks, employees often experience anxiety and uncertainty. Ma et al. (2026) found that GenAI provides immediate suggestions, reference solutions, and step-by-step guidance, which lowers the perceived difficulty of the task and alleviates anxiety. This reduction in negative emotional arousal fosters greater confidence in undertaking expanded role responsibilities. Collectively, these theoretical mechanisms and empirical findings indicate that GenAI use serves as a contextual enabler that bolsters employees’ confidence in their capacity to take on expanded work roles. Therefore, we propose:

H4: GenAI is positively correlated with employees’ role breadth self-efficacy.

Both social cognitive and role theorists agree that role breadth self-efficacy reflects a general willingness to transcend routine role boundaries and embrace expanded responsibilities. Individuals who believe in their ability to perform broader, proactive tasks are more willing to experiment with new technologies, integrate them into their workflows, and engage in joint problem-solving with intelligent systems (Syamsudin et al., 2022). Indeed, self-efficacy impacts the initiation, guidance, exertion, and tenacity of behavior (Bandura, 1986; Schunk, 1991). Empirical evidence supports that employees who possess this confidence tend to approach new work methods, including AI systems, as opportunities for role enhancement rather than threats to their existing job security (Syamsudin et al., 2022; Khan et al., 2024). They explained that employees with high self-efficacy tend to approach AI systems with curiosity and experimentation, viewing them as tools that extend their existing capabilities. In contrast, those with low self-efficacy may experience anxiety or avoidance when confronted with AI integration. Similarly, Hong (2022) suggested that those with increased role breadth efficacy view AI as more advantageous and productive, and tend to take advantage of its automation and enhancement capabilities. In total, employees with strong role breadth self-efficacy are more willing to experiment with AI tools, integrate them into workflows, and engage in joint problem-solving with intelligent systems. Thus, we assume that:

H5: Role breadth self-efficacy is positively correlated with employee-AI collaboration.

### 2.4 AI literacy

Role theory posits that environmental stimuli, such as the introduction of AI, reshape employees’ role perceptions (ambiguity and self-efficacy), which in turn influence their behavioral responses, including collaboration with AI. However, role theory primarily explains how individuals perceive and react to environmental changes, but it offers limited insight into why employees differ in how they interpret their roles when using generative AI, and why some employees successfully translate their role perceptions into constructive actions while others do not. Social cognitive theory suggests that the influence of the environment on individuals depends on their cognitive capacity to interpret and act upon their work environment. Among various individual difference variables that could potentially moderate the link, AI literacy is uniquely relevant to the present context. AI literacy directly addresses the knowledge and skills required to understand, interact with, and evaluate AI systems (Ng et al., 2021). It provides employees with the interpretive framework needed to making sense of AI-induced role changes and acting on their role perceptions effectively.

In a world in which algorithms are increasingly defining reality, the notion of literacy has been expanded. It is no longer limited to traditional abilities but now encompasses multiple areas of digital skills AI literacy covers an individual’s overall ability to understand and interact with artificial intelligence. This includes the ability to critically evaluate AI applications, the ability to express ideas clearly when using AI tools, and the ability to seamlessly collaborate with these systems as partners when solving problems. This is not only about technical knowledge, but also about cultivating wisdom to effectively respond to the AI-driven world (Long and Magerko, 2020). Wang et al. (2023) and Ng et al. (2021) proposed a structure for defining AI literacy, focusing on the skill to adeptly employ AI technologies (Usage), identify and understand AI technologies during interactions (Awareness), critically evaluate AI applications and their outputs (Evaluation), and the responsibilities related to AI use (Ethics). Similarly, Cetindamar et al. (2024) indicated that employees’ AI literacy can be subdivided into four key capabilities: technology-related (understanding the technology), ethical awareness-related (understanding ethical issues related to AI use), human-AI collaboration-related (optimizing teamwork with intelligent systems), and learning-related (concern regarding lifelong learning).

Role theory posits that environmental stimuli, such as the introduction of AI, reshape employees’ role perceptions (ambiguity or self-efficacy). However, role theory alone offers limited insight into why employees differ in how they interpret their roles when using GenAI. AI literacy can be understood as a critical cognitive resource that helps individuals navigate AI-augmented work environments by providing accurate mental models of AI’s capabilities and limitations (Zehui et al., 2025). This knowledge enables employees to clearly distinguish which tasks are appropriate for AI delegation and which must remain under their own control, thereby reducing role boundary ambiguity that might otherwise arise from unclear task allocation (Pinski et al., 2024; Lu and Lin, 2025). Moreover, AI literacy includes the ability to critically evaluate AI outputs. As Kulal (2025) found, rather than accepting AI-generated content uncritically, literate users engage in verification, validation, and adjustment. This critical engagement prevents cognitive offloading and skill atrophy, which in turn mitigates doubts about one’s own competence boundaries. In addition, AI literacy equips employees with the capacity to manage conflicts between AI recommendations and their own professional judgment. When discrepancies occur, literate users can understand the reasoning behind AI suggestions and make informed decisions, thus reducing role confusion stemming from such conflicts (Bewersdorff et al., 2025). Taken together, these findings imply that AI literacy may serve as a buffer that mitigates or counteracts employees’ role ambiguity when they use GenAI. Accordingly, we hypothesize:

H6: AI literacy moderates the negative relationship between GenAI and role ambiguity, that is, this relationship is weaker for employees with stronger AI literacy and stronger at lower levels.

AI literacy encompasses the ability to formulate precise prompts, engage in iterative dialogue with AI systems, and critically evaluate AI-generated outputs, which directly improve the quality of mastery experiences obtained from GenAI use (Wang et al., 2023). When employees possess the skills to refine prompts, interpret responses, and adjust their queries based on AI feedback, they are more likely to achieve successful task outcomes. These successful experiences serve as potent mastery experiences, which are the strongest driver of self-efficacy (Peng et al., 2026). Further, AI literacy also includes the capacity to extract transferable knowledge from AI-generated examples. By observing how GenAI structures solutions or generates novel ideas, employees can internalize new problem-solving strategies. This vicarious learning is more effective when the observer understands the underlying logic of AI outputs, which serves as a strong predictor of self-efficacy (Bewersdorff et al., 2025). Collectively, AI literacy strengthens the positive relationship between GenAI use and role breadth self-efficacy. Accordingly, we propose:

H7: AI literacy moderates the positive relationship between GenAI and role breadth self-efficacy, that is, this relationship is stronger for employees with stronger AI literacy and weaker at lower levels.

Table 1 provides an overview of the key constructs examined in this study, along with representative references and a brief description of each construct’s conceptual focus. These references were selected based on their foundational contributions, empirical relevance, and alignment with the measurement approaches adopted in the present research.

**TABLE 1:** Key studies and constructs in the proposed model.

Construct	Representative references	Key focus / measurement context
GenAI	Cheng et al., 2023; Zhang X. et al., 2025	Employees’ perceptions of using generative AI (e.g., ChatGPT, Copilot) for task-oriented purposes, including drafting, problem-solving, data synthesis, and knowledge acquisition; frequency and engagement with GenAI in daily work
Role ambiguity	Man Tang et al., 2022; Eatough et al., 2011	Role ambiguity in human-AI interaction; meta-analysis of role stressors; technostress and role ambiguity as a stressor from technology characteristics
Role breadth self-efficacy	Parker, 1998; Parker et al., 2006; Syamsudin et al., 2022	Conceptualization and measurement of RBSE; RBSE as antecedent of proactive behavior; RBSE and proactive work behavior
Employee-AI collaboration	Kong et al., 2023; Anthony et al., 2023; Chowdhury et al., 2022	Trust in AI and employee-AI collaboration; conceptualizing collaboration with AI; AI-employee collaboration and business performance
AI literacy	Schiavo et al., 2024; Wang et al., 2023; Long and Magerko, 2020	AI literacy, apprehension, and acceptance; AI literacy scale development; competencies and design considerations
Job performance	Williams and Anderson, 1991; Kong et al., 2023	In-role performance measurement; AI collaboration and performance outcomes

**FIGURE 1:**
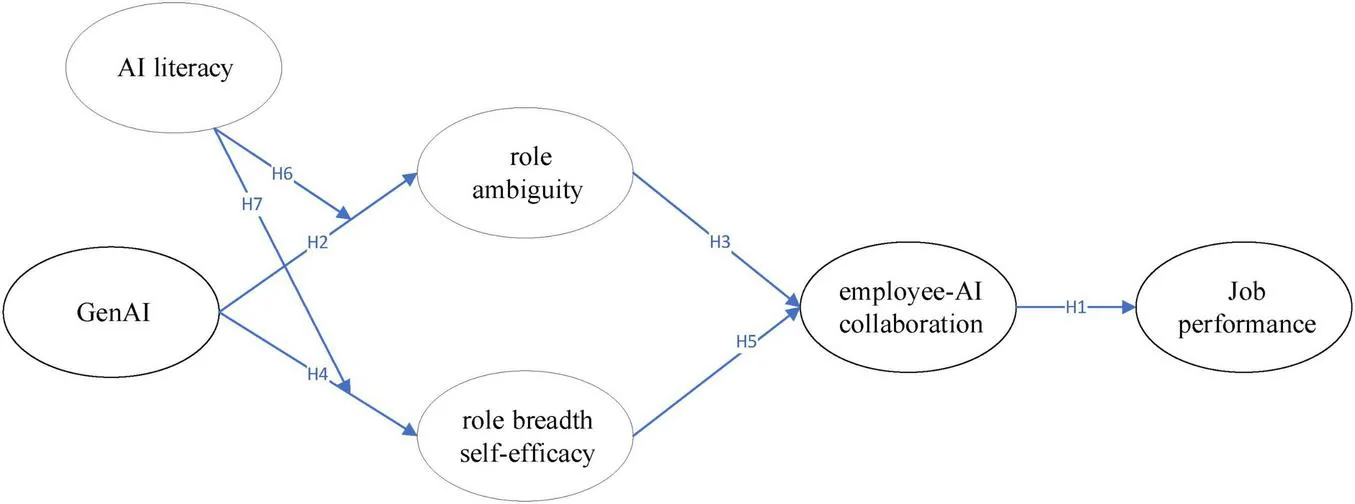
Conceptual model.

Building on the analysis, we developed the following conceptual framework (see Figure 1).

## 3 Materials and methods

### 3.1 Participants

This survey was conducted with the employees of five high-tech firms in Tianjin, China, which have experience using GenAI in their work or life. To facilitate participation, a QR code was shared with the respondents, enabling them to conveniently access the webpage and complete the questionnaire. At the beginning of the questionnaire, a detailed explanation was provided, clarifying that the research was centered on employees’ perceptions and reactions to GenAI.

In total, 580 participants comprised the research sample. Invalid responses were excluded based on the following criteria: (a) completion time less than 120 s (based on pilot test median completion time minus two standard deviations); (b) straight-lining responses (i.e., identical answers for more than 80% of consecutive items); (c) missing data for more than 10% of the core measurement items. Applying these criteria, 39 out of 580 responses were removed, yielding a final sample of 541 (49% female) valid cases.

The age distribution showed that 27.2%, 31.3%, 23%, 18.5% were below 25, 26–35, 35–46, and over 46 years, respectively. In the sample, 25.5%, 32.7%, 30.4%, and 11.5% had high school or lower, associate degrees, bachelor’s degrees, and master’s or higher, respectively. Management levels were distributed as follows: 63.4% non-managerial staff, 16.5% junior management, 11.3% middle management, 4.5% senior management, and 4.3% other. Regarding tenure, 27.2%, 31.3%, 23%, 15.4%, and 3.1% of employees had worked for less than 1 year, 1–2 years, 3–4 years, 5–6 years, and more than 6 years, respectively.

### 3.2 Methods

Where applicable and relevant, the items were adapted from existing literature. Considering the unique characteristics of the Chinese culture and organizational context, the original items were translated into Chinese through a comprehensive translation process that includes forward and reverse translation to ensure fidelity and accuracy of the intended meaning. Responses were scored on a 5-point Likert scale (1 = strongly disagree, 5 = strongly agree).

*Job performance*. Job performance was rated using a 7-item scale derived from Williams and Anderson (1991), to assess individuals’ work capabilities and performance outcomes. A sample item was “I can fulfil responsibilities specified in job description.” Cronbach’s alpha for the scale was 0.954.

*Employee-AI collaboration.* Employee-AI collaboration was rated using a 5-item scale, derived from Kong et al. (2023) to evaluate employees’ readiness to work with AI in decision-making, forecasting, issue resolution, data analysis, recognition of problems, identification of opportunities and risks. A sample item was “AI participates in my problem-solving process.” Cronbach’s alpha for the scale was 0.861.

*GenAI*. GenAI was rated using a 4-item scale, derived from Zhang X. et al. (2025), to assess employees’ use of GenAI. A sample item was “I use GenAI assistants to obtain ideas.” Cronbach’s alpha for the scale was 0.843.

*Role ambiguity.* Role ambiguity was rated using a 6-item scale, based on Man Tang et al. (2022), evaluating workers’ perceived role uncertainty. A sample item was “I do not know exactly what is expected of me.” Cronbach’s alpha for the scale was 0.886.

*Role breadth self-efficacy*. Role breadth self-efficacy was rated using a 3-item scale, derived from Man Tang et al. (2022) to assess employee confidence in their work roles. A sample item was “I am confident in helping to set goals at work.” Cronbach’s alpha for the scale was 0.835.

*AI literacy.* AI literacy was rated using an 8-item scale, derived from Schiavo et al. (2024), which assesses different aspects of AI literacy, such as AI utilization, comprehension, detection, and ethical considerations. A sample item was “I can assess the advantages and disadvantages associated with the use of AI.” Cronbach’s alpha for the scale was 0.894. The measurement scales used in this study are presented in Appendix 1.

## 4 Results

### 4.1 Descriptive statistics and correlations

Table 2 displays the summary statistics, including the means, standard deviations, composite reliabilities (CR), average variance extracted (AVE), and Pearson correlations for all latent variables. Composite reliabilities ranged from 0.895 to 0.962, all exceeding the recommended threshold of 0.70. AVEs ranged from 0.574 to 0.783, all above 0.5. The square root of each AVE (shown in bold on the diagonal) was larger than its correlations with any other construct, supporting both convergent and discriminant validity.

**TABLE 2:** Means, standard deviations, and correlations between the latent variables.

	Mean	SD	1	2	3	4	5	6	CR	AVE
1. GenAI	2.93	1.091	**(0.825)**						0.895	0.680
2. Role ambiguity	3.01	1.135	0.335***	**(0.799)**					0.914	0.638
3. Role breadth self-efficacy	2.98	1.144	0.360***	0.328***	**(0.867)**				0.901	0.752
4. Employee-AI collaboration	2.99	1.138	0.088*	−0.148***	0.180***	**(0.802)**			0.900	0.644
5. AI literacy	2.95	1.141	0.204***	0.243***	0.257***	0.037	**(0.757)**		0.915	0.574
6. Job performance	3.00	1.163	0.007	−0.110*	0.048	0.408***	−0.002	**(0.885)**	0.962	0.783

Bold figures on the diagonal are square roots of AVE. Correlations below the diagonal are Pearson correlations. CR, composite reliability; AVE, average variance extracted. *p < 0.05, ***p < 0.001.

As anticipated, GenAI correlated positively with role ambiguity (*r* = 0.335, *p* < 0.001) and with role breadth self-efficacy (*r* = 0.36, *p* < 0.001). Role ambiguity was negatively related to employee-AI collaboration (*r* = –0.148, *p* < 0.01), whereas role breadth self-efficacy showed a positive relationship with employee-AI collaboration (*r* = 0.18, *p* < 0.001). Moreover, employee-AI collaboration was positively associated with job performance (*r* = 0.408, *p* < 0.001). These results offer initial support for our hypotheses.

### 4.2 Test of the measurement model

We conducted a series of confirmatory factor analyses (CFA) to examine the discriminant validity of the six core constructs (GenAI, role ambiguity, role breadth self-efficacy, employee-AI collaboration, job performance, and AI literacy). The hypothesized six-factor model was compared against two alternative models: a five-factor model in which role ambiguity and role breadth self-efficacy were combined into one factor, and a three-factor model that further merged AI literacy with the above two factors and also combined employee-AI collaboration with job performance. The fit indices for all models are summarized in Table 3.

**TABLE 3:** Measurement model result.

Model	χ ^2^	*df*	RMSEA	CFI	SRMR
Six-factor	561.94	480	0.018	0.992	0.03
Five-factor	1599.69	486	0.065	0.889	0.107
Three-factor	4670.48	495	0.125	0.583	0.199

The six-factor model demonstrated an excellent fit to the data: χ^2^(480) = 561.94; CFI = 0.992; RMSEA = 0.018; SRMR = 0.03. All indices met or exceeded the recommended thresholds (CFI > 0.95, RMSEA < 0.05, SRMR < 0.08), indicating that the measurement structure was well supported. The five-factor model showed a marginal fit (CFI = 0.889, RMSEA = 0.065, SRMR = 0.107), and its chi-square difference relative to the six-factor model was highly significant (Δχ^2^(6) = 1037.75, p < 0.001). The three-factor model yielded a very poor fit (CFI = 0.583, RMSEA = 0.125, SRMR = 0.199). These results confirm that the six distinct factors are empirically distinguishable and that the measurement model is appropriate for subsequent hypothesis testing.

### 4.3 Testing the hypotheses

We tested our hypotheses using Bayesian estimation with Mplus 8.3. The potential scale reduction (PSR) values were below 1.05 after the final iteration, indicating satisfactory convergence. Table 4 shows the results of hypotheses paths.

**TABLE 4:** Structural model.

Hypothesis and relationship	β -value	95%CI	outcomes
Panel A. Main effects			
GenAI→RA	0.347	[0.272, 0.415]	H2 supported
GenAI→RSE	0.347	[0.273, 0.414]	H4 supported
RA→Collab	−0.232	[−0.314, −0.147]	H3 supported
RSE→Collab	0.256	[0.170, 0.338]	H5 supported
Collab→JP	0.409	[0.335, 0.477]	H1 supported
Panel B. Moderating effects			
GenAI × AIL→RA	−0.204	[−0.277, −0.128]	H6 supported
GenAI × AIL→RSE	0.168	[0.092, 0.242]	H7 supported

RA, Role ambiguity; RSE, Role breadth self-efficacy; Collab, Employee-AI collaboration; JP, Job performance; AIL, AI literacy.

The results showed that GenAI was positively related to role ambiguity (β = 0.347, 95% CI [0.272, 0.415]) and to role breadth self-efficacy (β = 0.347, 95% CI [0.273, 0.414]), supporting H2 and H4, respectively. Role ambiguity had a negative effect on employee-AI collaboration (β = –0.232, 95% CI [–0.314, –0.147]), while role breadth self-efficacy had a positive effect (β = 0.256, 95% CI [0.170, 0.338]), thus supporting H3 and H5. Employee-AI collaboration was positively associated with job performance (β = 0.409, 95% CI [0.335, 0.477]), confirming H1.

We also examined the moderating role of AI literacy. The interaction between GenAI and AI literacy on role ambiguity was negative and significant (β = –0.204, 95% CI [–0.277, –0.128]), indicating that high AI literacy attenuates the positive association between GenAI and role ambiguity, thus supporting H6. Likewise, the interaction between GenAI and AI literacy on role breadth self-efficacy was positive and significant (β = 0.168, 95% CI [0.092, 0.242]), suggesting that AI literacy strengthens the association between GenAI and role breadth self-efficacy, supporting H7.

**FIGURE 2:**
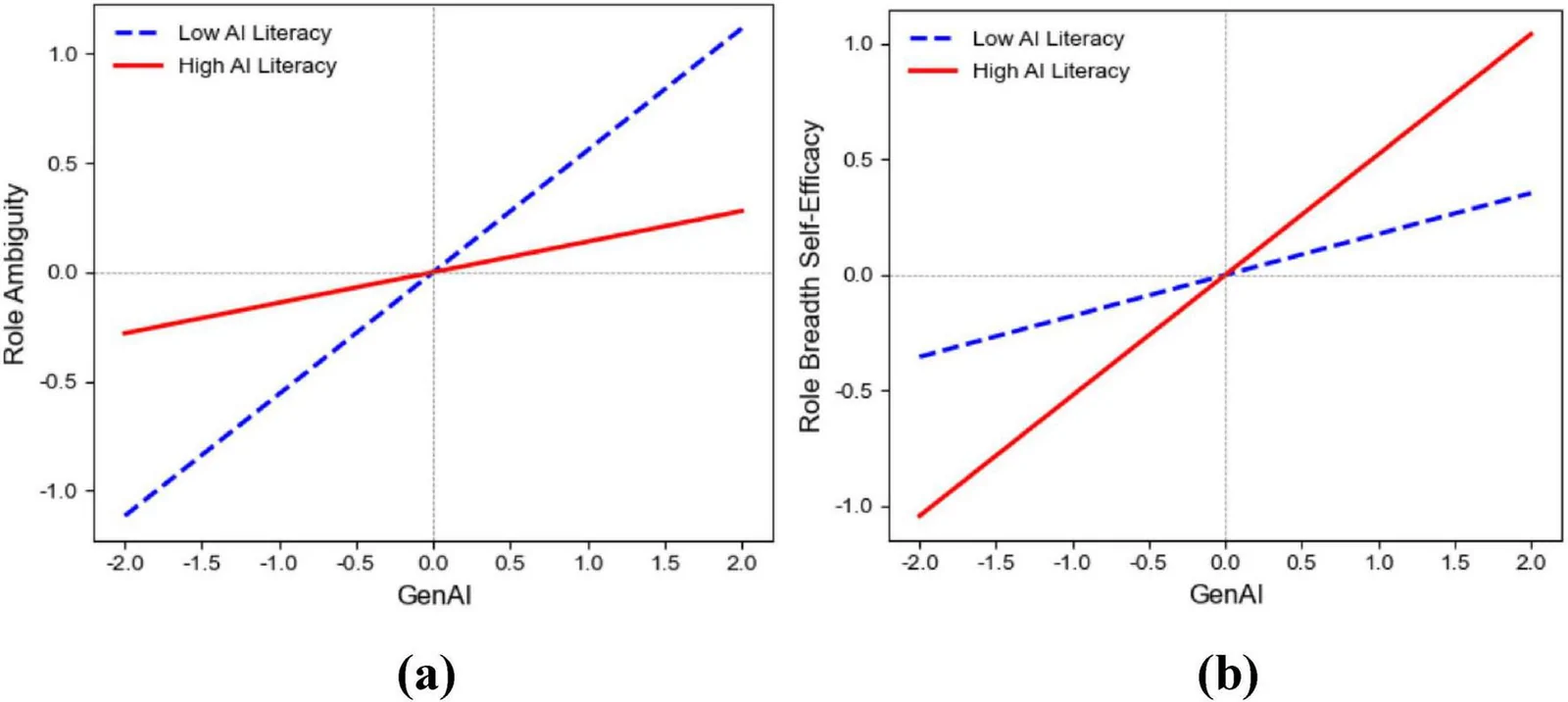
Moderating effect of AI literacy. **(a)** Moderating effect of AI literacy on the relationship between role ambiguity and employee-AI collaboration. **(b)** Moderating effect of AI literacy on the relationship between role breadth self-efficacy and employee-AI collaboration.

To interpret the interactions, we performed simple slope analyses at one standard deviation below and above the mean of AI literacy. Figure 2a presents the results for the moderating effect of AI literacy on the relationship between GenAI and role ambiguity. It shows that, at low levels of AI literacy (β = 0.558, 95% CI [0.441, 0.673]), the positive relationship was significant, whereas at high levels of AI literacy (β = 0.140, 95% CI [0.032, 0.248]), the relationship became weaker and only marginally significant (with the confidence interval approaching zero). This indicates that high AI literacy attenuates the positive effect of GenAI on role ambiguity. Figure 2b shows that the relationship between GenAI and role breadth self-efficacy was positive at both low and high levels of AI literacy. This positive effect was significantly stronger at high AI literacy (β = 0.521, 95% CI [0.415, 0.626]) than at low AI literacy (β = 0.177, 95% CI [0.064, 0.293]), confirming that AI literacy enhances the positive influence of GenAI on role breadth self-efficacy.

## 5 Discussion

This study aimed to uncover how GenAI relates to employees’ perceived collaboration with AI systems, drawing on role theory. We proposed a dual-path model in which GenAI relates to employee-AI collaboration through role ambiguity and role breadth self-efficacy, with AI literacy as a moderator. Analyzing survey data from 541 employees in Chinese high-technology firms, we found that GenAI is positively associated with both role ambiguity and role breadth self-efficacy. Role ambiguity is negatively associated with employee-AI collaboration, whereas role breadth self-efficacy is positively associated with it. More importantly, AI literacy significantly moderates both relationships: at high levels of AI literacy, the positive association between GenAI and role ambiguity is significantly attenuated, while the positive association between GenAI and role breadth self-efficacy is strengthened. Employee-AI collaboration, in turn, is associated with enhanced job performance. These findings offer several theoretical and practical contributions.

### 5.1 Theoretical implications

First, this study provides a dual-path model that captures both the disruptive and enabling effects of GenAI on employees’ role perceptions, thereby extending role theory to the context of human–AI collaboration. Prior research has largely focused on AI’s technical capabilities or employees’ general attitudes toward GenAI (Tambe et al., 2019). However, these studies have overlooked the role-based cognitive mechanisms that mediate the relationship between GenAI use and collaboration intentions. Our findings show that GenAI simultaneously increases role ambiguity and role breadth self-efficacy. This duality mirrors the automation-augmentation paradox identified by Raisch and Krakowski (2021) and extends it by demonstrating that both pathways significantly affect collaboration intentions. This finding suggests that GenAI should not be viewed as uniformly positive or negative; its impact depends on which role perception predominates and how employees interpret AI-induced changes. By uncovering this dual-pathway mechanism, our study answers the call for more nuanced understanding of how AI shapes employees’ psychological states and behavioral intentions (Cheng et al., 2023).

Second, this study clarifies the role ambiguity pathway and identifies AI literacy as a critical boundary condition that weakens this hindrance mechanism. The significant positive effect of GenAI on role ambiguity aligns with classic role theory (Lee and Ashforth, 1996; Eatough et al., 2011) and prior studies on role stressors in technology contexts (van Slooten et al., 2024; Mañas et al., 2018). However, our study goes beyond these findings by demonstrating that this relationship is not fixed. Specifically, the moderating effect of AI literacy reveals that role ambiguity is not an inevitable consequence of GenAI use; rather, it depends on employees’ ability to understand and interact with AI systems. This finding extends previous research that treated AI literacy primarily as a direct antecedent of AI acceptance or performance (Wang et al., 2023; Schiavo et al., 2024). Instead, we position AI literacy as a cognitive resource that helps employees distinguish between AI’s capabilities and their own responsibilities, thereby reducing the role ambiguity triggered by GenAI use. This theoretical insight answers the question of why some employees experience role ambiguity when using GenAI while others do not.

Third, this study clarifies the role breadth self-efficacy pathway and identifies AI literacy as a conditioner that strengthens this enabling mechanism. The positive effect of GenAI on role breadth self-efficacy supports prior work on proactive behavior (Parker et al., 2006; Syamsudin et al., 2022) and AI self-efficacy (Hong, 2022; Khan et al., 2024). However, our study is among the few to demonstrate that GenAI use can enhance role breadth self-efficacy, and that this effect is stronger when employees possess higher AI literacy. This finding is consistent with social cognitive theory (Bandura, 1986), which emphasizes that mastery experiences are more effective when individuals have the relevant cognitive skills to interpret and learn from these experiences. In our context, AI-literate employees are better positioned to obtain mastery experiences from successful GenAI use, thereby amplifying their confidence in taking on broader responsibilities. This theoretical insight advances our understanding of how GenAI can serve as an enabling technology that empowers employees to expand their role capabilities, rather than merely automating their tasks.

Fourth, the distinct moderating roles of AI literacy on the two pathways reveal a nuanced picture. Consistent with existing evidence (Högemann et al., 2025; Li et al., 2025; Sun C. et al., 2025), we find that AI literacy weakens GenAI’s positive effect on role ambiguity while strengthening its positive effect on role breadth self-efficacy. This dual moderating effect underscores that AI literacy is not merely a general competence but a critical cognitive resource that shapes how employees interpret and respond to GenAI-induced role changes (Yin et al., 2025). By bridging role theory and social cognitive theory, our study establishes AI literacy as a key boundary condition that determines whether GenAI’s influence tilts toward disruption or empowerment.

### 5.2 Practical implications

Our findings offer several practical insights for organizations implementing AI. First, as GenAI inevitably increases role ambiguity, organizations should proactively manage role clarity. However, our results show that simply clarifying roles may not be sufficient; instead, investing in AI literacy training can help employees reinterpret ambiguity constructively. Specifically, when employees understand AI’s capabilities and limitations, they are more likely to view ambiguous role expectations as opportunities for role expansion rather than as threats. Thus, organizations should combine role clarification with AI literacy programs that cover technical, ethical, and collaborative aspects of AI use (Long and Magerko, 2020; Cetindamar et al., 2024).

Second, role breadth self-efficacy can be seen as a valuable personal resource. Yet, low AI literacy can render generative AI use less effective in boosting self-efficacy. Organizations should not only encourage proactive role behaviors (e.g., through job enrichment or participative goal setting) but also provide hands-on AI training that builds employees’ confidence in using AI tools. Co-design workshops, where employees participate in selecting or customizing AI applications, can simultaneously enhance self-efficacy and AI literacy.

Third, employee-AI collaboration directly improves job performance. Hence, managers should design workflows that genuinely integrate AI as a collaborative partner rather than a replacement. Performance evaluation systems should reward effective human-AI collaboration, not just individual task completion.

## 6 Limitations and future research

Although this study advances our understanding of human-AI collaboration, several limitations need attention. First, this study focused on general-purpose generative AI tools (e.g., ChatGPT, Copilot) that employees use voluntarily for task assistance. Other forms of GenAI, such as domain-specific agents, highly autonomous systems, or mandatorily deployed tools, may affect role perceptions differently. Second, although role theory and social cognitive theory provide a robust foundation, this framework does not fully account for organizational contextual factors that may define human-AI collaboration outcomes. Organizational structures (e.g., centralized versus decentralized decision-making), cultural norms (e.g., innovation climate and risk tolerance), and resource allocation strategies (e.g., AI training budgets) likely moderate how employees perceive and adapt to AI-driven role change (Bakir et al., 2025). Future studies can examine how organizational contextual factors interact with individual factors that affect employee-AI collaboration. Therefore, the generalizability of our findings to other AI types requires further investigation. Third, the cross-sectional data from Chinese high-technological firms limits causal inferences and generalizability. Future research should test this conceptual framework based on different cultural factors to improve its universality. Fourth, although we collected data from multiple firms to enhance generalizability, all variables were self-reported by the same respondents at the same time, which may introduce common method bias (Podsakoff et al., 2012). To assess this risk, we took several procedural remedies, including ensuring anonymity and confidentiality, using established scales with clear instructions, and separating measurement of independent and dependent variables in the questionnaire. However, we acknowledge this limitation and encourage future research to use multi-source data (e.g., supervisor-rated job performance) or experimental designs to strengthen causal inference.

## 7 Conclusion

This study examined how GenAI is associated with employee-AI collaboration through two parallel mechanisms, and how AI literacy moderates these pathways. These findings extend role theory by portraying GenAI as both a disruptor (increasing role ambiguity) and an enabler (enhancing role breadth self-efficacy), with AI literacy serving as a critical boundary condition that determines how employees form and interpret their role perceptions when using GenAI. Organizations should not only clarify role boundaries and foster self-efficacy through participatory design but also invest in AI literacy training to help employees interpret ambiguous situations constructively and leverage AI effectively.

## Data Availability

The raw data supporting the conclusions of this article will be made available by the authors, without undue reservation.

## Appendix

**Appendix A1:** Measurement

Constructs	Measurements
GenAI use	1. I use GenAI assistants to obtain ideas 2. I use GenAI assistants to acquire solutions for problems 3. I use GenAI assistants to get knowledge 4. I use GenAI assistants to ask questions
Employee-AI collaboration	1. AI participates in my decision-making process 2. AI participates in my prediction process 3. AI participates in my problem-solving process. 4. AI participates in my information identification and evaluation process. 5. AI participates in my problems, opportunities, or risk recognition process.
Role ambiguity	1. I don’t have clear, planned goals and objectives for my job 2. I don’t know whether I have divided my time properly. 3. I don’t know exactly what is expected of me. 4. I don’t know what my responsibilities are. 5. The explanation of what has to be done is unclear. 6. I feel uncertain about how much authority I have.
Role breadth self-efficacy	1. I am confident in helping to set goals at work 2. I am confident in presenting information to a group of colleagues 3. I am confident in designing new procedures for my work area
AI literacy	1. I know the definitions of AI. 2. I can assess what advantages and disadvantages the use of an AI entails. 3. I can imagine possible future uses for AI. 4. I can operate AI applications in everyday life 5. In everyday life, I can work together gainfully with an artificial intelligence 6. I can tell if I am dealing with an application based on Artificial Intelligence. 7. I can distinguish whether I interact with an AI or a “real human”. 8. I can incorporate ethical considerations when deciding whether to use data provided by AI.
Job performance	1. Adequately completes assigned duties 2. Fulfills responsibilities specified in job description 3. Performs tasks that are expected of him 4. Meets formal performance requirements of the job 5. Engages in activities that will directly affect his performance evaluation 6. Neglects aspects of the job he is obligated to perform (R) 7. Fails to perform essential duties (R)
